# Encapsulating Transition Metal Nanoparticles inside Carbon (TM@C) Chainmail Catalysts for Hydrogen Evolution Reactions: A Review

**DOI:** 10.3390/molecules29194677

**Published:** 2024-10-02

**Authors:** Jiamin Zhao, Meimei Kou, Qing Yuan, Ying Yuan, Jinsheng Zhao

**Affiliations:** School of Chemistry and Chemical Engineering, Liaocheng University, Liaocheng 252059, China; zhaojiamin08031223@163.com (J.Z.); kmm2018705344@163.com (M.K.); yuanqing@lcu.edu.cn (Q.Y.)

**Keywords:** hydrogen energy, HER, TM@C, chainmail catalyst

## Abstract

Green hydrogen energy from electrocatalytic hydrogen evolution reactions (HERs) has gained much attention for its advantages of low carbon, high efficiency, interconnected energy medium, safety, and controllability. Non-precious metals have emerged as a research hotspot for replacing precious metal catalysts due to low cost and abundant reserves. However, maintaining the stability of non-precious metals under harsh conditions (e.g., strongly acidic, alkaline environments) remains a significant challenge. By leveraging the curling properties of two-dimensional materials, a new class of catalysts, encapsulating transition metal nanoparticles inside carbon (TM@C) chainmail, has been successfully developed. This catalyst can effectively isolate the active metal from direct contact with harsh reaction media, thereby delaying catalyst deactivation. Furthermore, the electronic structure of the carbon layer can be regulated through the transfer of electrons, which stimulates its catalytic activity. This addresses the issue of the insufficient stability of traditional non-precious metal catalysts. This review commences with a synopsis of the synthetic advancement of the engineering of TM@C chainmail catalysts. Thereafter, a critical discussion ensues regarding the electrocatalytic performance of TM@C chainmail catalysts during hydrogen production. Ultimately, a comprehensive review of the conformational relationship between the structure of TM@C chainmail catalysts and HER activity is provided, offering substantial support for the large-scale application of hydrogen energy.

## 1. Introduction

Electrocatalytic hydrogen evolution reactions (HERs) from water are an efficient approach for producing green hydrogen on a large scale due to its advantages of low carbon, high efficiency, and controllability [1,2,3,4,5]. At present, the most effective catalysts for HERs are those based on precious metals, with platinum-based catalysts being particularly effective [6,7,8,9,10,11,12]. However, the utilization of precious metal catalysts is constrained by several significant disadvantages, including high cost and limited reserves, which restrict their practical applications [13,14,15,16,17]. Thus, in recent years, non-precious metals have emerged as a prominent area of research interest, with their potential to replace precious metal catalysts [18,19,20,21,22]. This has been largely driven by the favorable cost and availability characteristics of non-precious metals [23,24,25]. However, maintaining the stability of non-precious metals under harsh conditions (e.g., strongly acidic and alkaline environments) remains a challenge [26,27]. 

To address this problem, an advanced solution employs the use of carbon layers to encapsulate three-dimensional transition metal nanoparticles [28,29,30]. In carbon-encapsulated catalysts (TM@C chainmail catalysts), the transfer from the inner metal to the carbon layer allows for the effective modulation of the electronic density of the carbon layer, thereby stimulating HER catalytic activity on the surface of the carbon layer. Furthermore, the carbon layer on the inner metals prevents direct contact with harsh reaction environments (e.g., strong acids, strong bases, etc.), effectively delaying the deactivation of the catalyst. The distinctive benefits of carbon-coated catalysts can be leveraged to enhance the resilience of transition metal catalysts in challenging environments. Due to their unique advantages, TM@C chainmail catalysts have been increasingly used in recent years in a number of important research areas (e.g., fuel cells, CO_2_ conversion, solar cells, and metal-air batteries), where their performance is not only comparable to that of precious metals but also has excellent stability [31,32,33,34,35].

This work presents recent progress in the application of HER electrocatalysts utilizing TM@C chainmail catalysts. The initial section of the paper considers the methods used to prepare carbon chainmail layers on the surface of inner metals. Second, a critical discussion about the electrocatalytic performance of TM@C chainmail catalysts during hydrogen evolution reactions (HERs) is presented. The objective of this section is to identify the principal active sites of TM@C chainmail catalysts in order to ascertain the optimal means of enhancing catalyst activity. Finally, a comprehensive review of the conformational relationship between the structure of TM@C chainmail catalysts and HER performance is presented at the atomic, molecular, and macroscopic levels. 

## 2. Preparation Methods of TM@C Chainmail Catalysts

**Figure 1:**
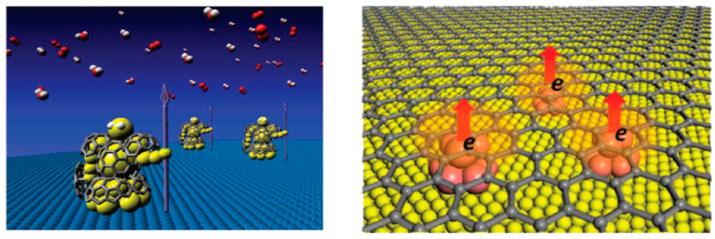
Schematic structure of typical carbon-coated metal nanomaterials [38]. Reproduced with permission from ref. [31]. Copyright 2017, Wiley.

TM@C chainmail catalysts comprise a core of metal nanoparticles and several layers of graphitized carbon tightly wrapped around the periphery of the metal particles (Figure 1). In 1993, Ruoff et al. [36] employed the arc discharge method to identify LaC_2_@C chainmail catalysts for the first time, with an average particle size of 20–40 nm. They also demonstrated, through experimentation, that carbon shells can effectively safeguard LaC_2_ from degradation. The TM@C chainmail catalysts represent a novel class of materials that can effectively safeguard the coated metal core, thereby offering a promising avenue for the broader deployment of air-sensitive nanomaterials. Subsequently, TM@C chainmail catalysts have garnered significant interest from the research community. In addition to the arc discharge method, two further methods have been developed for the preparation of TM@C chainmail catalysts. These are chemical vapor deposition and pyrolysis [11,28,29,37].

### 2.1. Chemical Vapor Deposition

Chemical vapor deposition (CVD) has been employed extensively in the synthesis of carbon-based materials including carbon nanorods, carbon fiber, carbon nanotubes, graphene, and carbon-covered metal nanoparticles [39,40]. The initial step involves the introduction of the metal or oxide to be coated onto the carrier into the constant temperature zone of the reaction chamber. This is followed by an increase in temperature and the introduction of the carbon source, which is pyrolyzed by the catalytic effect of the metal or oxide at a specific temperature [41,42,43]. This process results in the production of carbon and its crystallization into graphite. The final stage involves the formation of carbon-coated metal nanomaterials. The gas-phase carbon sources employed in the preparation of carbon-coated metal nanomaterials via CVD are predominantly small-molecule organic compounds, including methane (CH_4_), pyridine, acetonitrile, and ethylene. 

**Figure 2:**
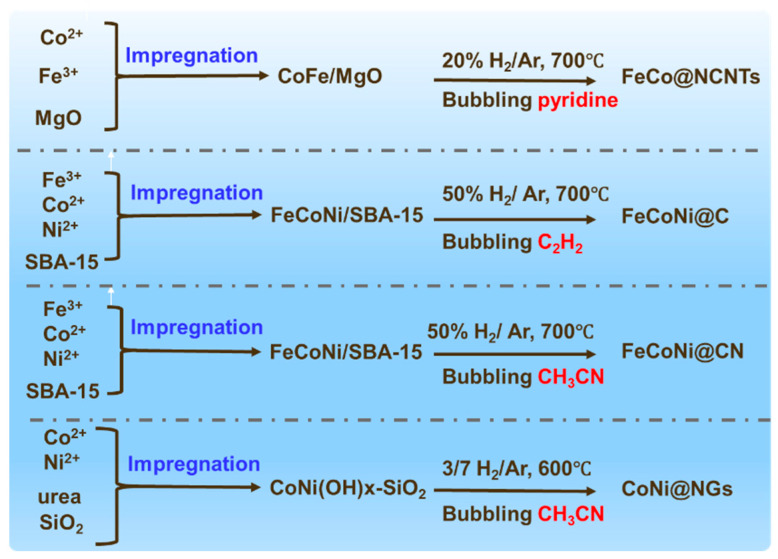
A simple summary of CVD-synthesis TM@C.

Based on the CVD approach, Deng et al. [37] synthesized nitrogen-rich, carbon-nanotube-coated iron–cobalt alloy nanomaterials (FeCo@NCNTs-NH) through the use of iron and cobalt salts as precursors, pyridine as a carbon source, and ammonia as a nitrogen source. The HER onset overpotential was observed to be a mere 70 mV under acidic conditions, a value that was found to be in close proximity to that of 40% of commercial Pt/C catalysts. Then, they [11] prepared nitrogen-rich, graphene-coated iron–nickel alloy nanomaterials using iron and nickel salts as precursors and acetonitrile as the carbon and nitrogen sources. The η_10_ of OER was found to be only 280 mV under alkaline conditions, and the material demonstrated good stability after 10,000 cycles. Shen et al. [44] prepared carbon-coated nickel–copper alloy nanomaterials (NiCu@C) with nickel and copper salts as precursors and methane as a carbon source. These materials exhibited good HER activity and stability, with an η10 of 48 mV under acidic conditions and 74 mV under alkaline conditions. A concise overview of the CVD synthesis of TM@C is presented in Figure 2.

Moreover, organometallic sources can be employed as direct precursors to synthesize carbon-coated nanomaterials via thermal decomposition and autocatalysis. Wang et al. [45] prepared carbon-coated copper nanomaterials through a process involving the heating and evaporation of acetylacetone, which was then deposited by pyrolysis under a hydrogen atmosphere. Deng et al. [46] employed SBA-15 as a sacrificial carrier, impregnated metal iron, cobalt, and nickel salts with hydrogen, deposited them with acetonitrile gas, and subsequently removed the templates and incomplete particles through HF etching to prepare carbon-coated nanomaterials with different transition metals. 

The advantages of the CVD method for carbon-coated materials are as follows: it is straightforward to operate, simple to control the coating material, and straightforward for producing large batches. However, it also has its inherent disadvantages: the necessity to introduce a gas-phase carbon source, low operational safety, and the tendency of metal particles to agglomerate (for example, in the process of synthesizing metal alloys, high-temperature hydrogen reduction is required before capping).

### 2.2. Pyrolysis Method

Pyrolysis is a method of preparing carbon-coated metal nanoparticles through direct pyrolysis of mixtures or metal–organic compounds containing metal salts and organic matter, whereby the carbon source is provided under an inert atmosphere without the addition of a gas-phase carbon source [47]. The pyrolysis method is typically classified into two categories based on the type of metal salt and organic matter employed. The first category encompasses the metal–organic complex-assisted pyrolytic carbonization method. The second is the pyrolytic carbonization of metal–organic framework (MOF) materials. The following paragraphs provide a brief overview of the two aforementioned pyrolysis methods.

#### 2.2.1. Metal–Organic Complex-Assisted Pyrolytic Carbonization Method

**Figure 3:**
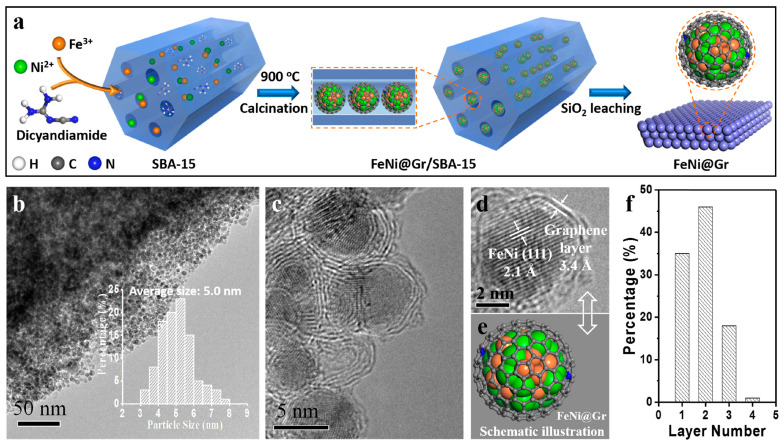
(**a**) The preparation route for FeNi@ chainmail catalysts, (**b**–**d**) TEM image of FeNi@ chainmail catalysts, (**f**) the carbon layers of FeNi@ chainmail catalysts [48]. Reproduced with permission from ref. [48]. Copyright 2018, Elsevier.

Metal–organic complex-assisted pyrolysis is a method of preparing carbon-coated metal nanoparticles through the pyrolysis and carbonization of a metal–organic complex under the catalytic action of a metal center. The most commonly utilized organic compounds are dicyandiamine, melamine, and urea, which are capable of producing melamine at elevated temperatures. The metal salts may be either inorganic or organometallic. To illustrate, the powder produced from a combined solution of nickel salt, iron salt, and dicyandiamide was evaporated into a tube furnace and pyrolyzed under an argon atmosphere. This process resulted in the formation of carbon-encapsulated nickel-iron alloy nanomaterials, as demonstrated in Figure 3 [48]. By changing the thermal temperature and ratio of encapsulated FeNi metal, it is feasible to enhance the electronic density of the FeNi@graphene surface, thus attaining equilibrium in the adsorption strength of H* and OH*, where * represents the catalyst surface. The catalyst produced under optimal conditions exhibits an OER of η_10_ of only 280 mV, which is superior to that of commercial IrO_2_. Asefa et al. [49] prepared nitrogen-rich carbon-coated molybdenum carbide nanomaterials (Mo_2_C@NC) using direct mixing of ammonium molybdate with dicyandiamide, followed by high-temperature calcination under an argon atmosphere in a tube furnace. The Mo_2_C@NC demonstrated remarkable catalytic activity across a broad pH range, exhibiting an η_10_ of 60 mV in acidic and alkaline conditions and an η_10_ of 156 mV in neutral conditions. Furthermore, it exhibited near-100% Faraday efficiency towards the HER. Subsequently, Asefa et al. [50] synthesized a Co@NC chainmail catalyst through the homogeneous mixing of dicyandiamide and cobalt salts, followed by their placement in a tube furnace under high temperatures and a nitrogen atmosphere. This was conducted in two stages, with the powders undergoing calcination for 2 h at 500 °C and 700 °C, respectively. Liu et al. [51] placed a powder prepared by evaporating a mixed solution of nickel salt, iron salt, and dicyandiamide into a tube furnace and calcined it at 800 °C under a nitrogen atmosphere. They then conducted a systematic investigation into the effect of one metal on the other as an additive, varying the ratio of added nickel salt to iron salt. It was demonstrated that a Ni/Fe ratio of 1:1 exhibited the most optimal catalytic effect.

Ƞ

#### 2.2.2. Pyrolytic Carbonization of Metal–Organic Framework Materials

The pyrolysis of metal–organic frameworks (MOFs) in an inert or reducing atmosphere can directly prepare carbon-coated metal nanomaterials, which is one of the most active research areas in this field in recent years [52,53,54]. This is due to the fact that the organic ligands in MOF materials can be used as carbon sources, and the metal ion nodes, which are uniformly dispersed at the atomic level, can be used as metal precursors. Xu et al. [55] developed a facile preparation of non-precious metal bifunctional electrolytic water catalysts, and nickel-based MOF materials were synthesized by ligand self-service assembly of Ni(NO_3_)_2_ as a metal source and triethylenediamine and terephthalic acid as ligands in N,N-dimethylformamide solution. Then, the nitrogen-doped carbon-coated nickel nanomaterials (Ni@NC) were prepared by calcining the prepared MOF-Ni in the range of 600 to 900 °C for 2 h under a nitrogen atmosphere. Zhang et al. [56] synthesized ZIF-67 by ligand self-assembly in methanol solution using CoCl_2_ as the metal source, polyvinylpyrrolidone as the active agent, and 2-methylimidazole as the ligand. The ZIF-67 powder was thoroughly mixed with an aqueous solution of H_3_BO_3_ and evaporated to dryness, and then the mixture was placed under an argon atmosphere in a tube furnace in two steps of calcination at 450 °C for 2 h. Following an incubation period of one hour at 750 °C, the ZIF-67 skeleton was observed to undergo a collapse, resulting in the formation of new components. In parallel with the dissolution of amorphous carbon into cobalt nanoparticles, surface diffusion of N and B atoms was observed and finally assembled into N- and B-co-doped Co@C chainmail catalysts.

The pyrolysis device is straightforward to operate and does not necessitate the passage of flammable and explosive gases, such as hydrocarbons, as carbon sources, thus ensuring enhanced safety. The synthesis of the carbon-capping material occurs in situ during the preparation process, and the metal particles can be nanosized, reducing the likelihood of agglomeration. This facilitates the production of large volumes of material. Nevertheless, pyrolysis also has inherent limitations. Cyanamide ligands are susceptible to decomposition, graphitization efficiency is low, and large quantities are required. Additionally, the inability to promote the formation of metal centers at the atomic level of dispersion results in a wide range of particle size distributions in the final product. Furthermore, metal–organic framework materials are complex to prepare, with low yields, and require a significant number of organic solvents. Notwithstanding these limitations, pyrolysis continues to represent a significant and widely used approach for the synthesis of carbon-coated metal nanoparticles.

## 3. Application in HER

**Figure 4:**
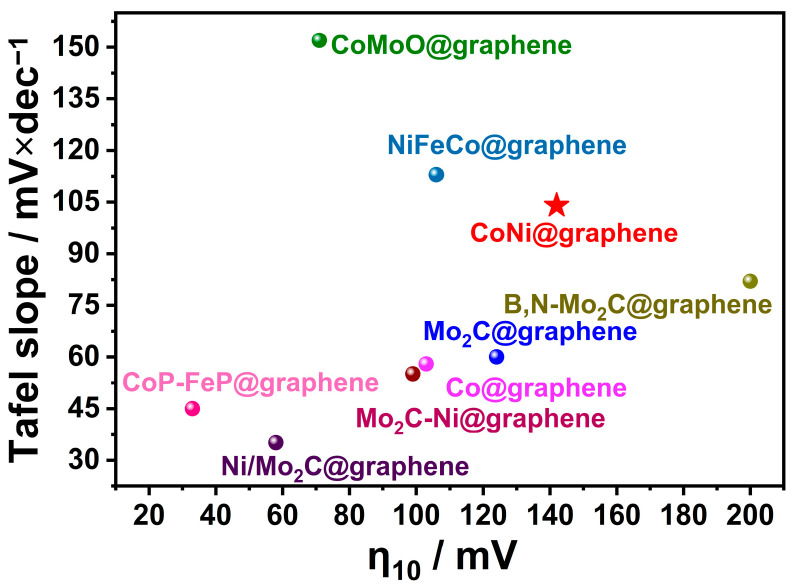
Summary of recent TM@graphene chainmail catalysts for HERs (detailed information is shown in Supporting Information Table S1).

The benefits of these TM@C chainmail catalysts make them suitable for use in improving stability in challenging environments, including strong alkaline or acidic media, high overpotentials, and high temperatures. A summary of recent TM@graphene catalysts for HERs is shown in Figure 4. This review can be divided into three sections based on the type of chainmail catalyst: transition metal alloy chainmail catalysts, transition metal carbide chainmail catalysts, and noble metal-doped transition metal chainmail catalysts.

### 3.1. Transition Metal Alloy Chainmail Catalysts 

**Figure 5:**
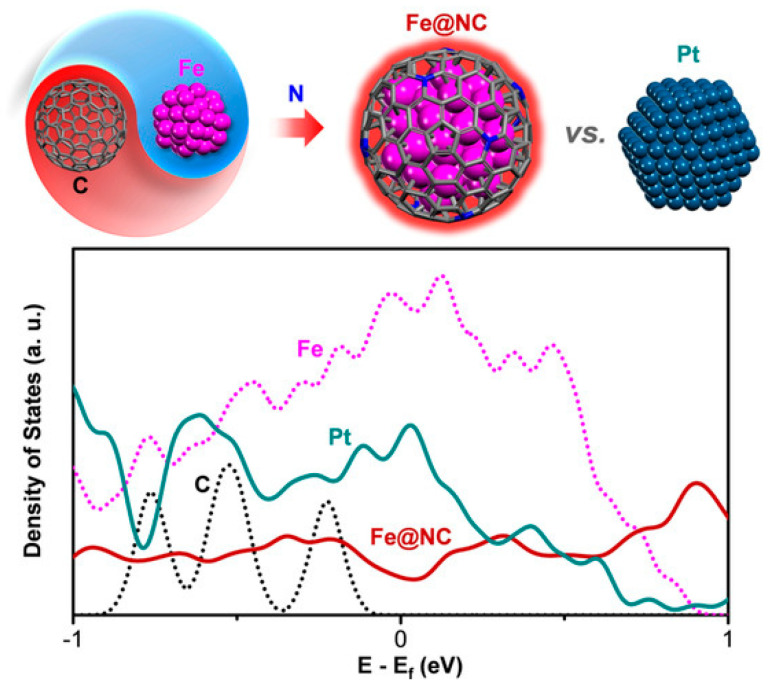
The density of states of Fe@NC and Pt NPs [57]. Reproduced with permission from ref. [57]. Copyright 2015, Wiley.

It is widely acknowledged that hydrogen adsorption on graphene is relatively weak. This is primarily due to the fact that the graphene surface is chemically inert, which results in a thermodynamically unfavorable adsorption process. Consequently, the HER activity of graphene is markedly inferior. Following the coating of transition metal (TM) nanoparticles with graphene, electrons are transferred from the TMs to the graphene due to the lower work function of TMs in comparison to that of graphene [34]. Electronic states in the vicinity of the Fermi energy level of carbon atoms are markedly altered, resulting in the energy band centers of the C-H bond-occupied states being situated at lower energy levels. This consequently strengthens the chemical bonding between hydrogen atoms and carbon atoms, thereby enhancing the adsorption of hydrogen on the surface of graphene and increasing the HER activity. Concomitantly, the stability of non-precious metal catalysts is markedly enhanced as a consequence of the protective effect of the graphene shell. Additionally, the combination of the electrical states of the chainmail carbon layers and the inner metallic cores can be exploited to replicate the electronic structures of precious metal catalysts, such as Pt, thereby replicating their HER catalytic abilities (Figure 5). This has been presented to be a practical method, wherein chain-mail catalysts consisting of a few-layer or single-layer graphene shells encapsulating three-dimensional metal nanoparticles have been found to surpass the HER activity of precious metal catalysts such as Pt [8,57].

**Figure 6:**
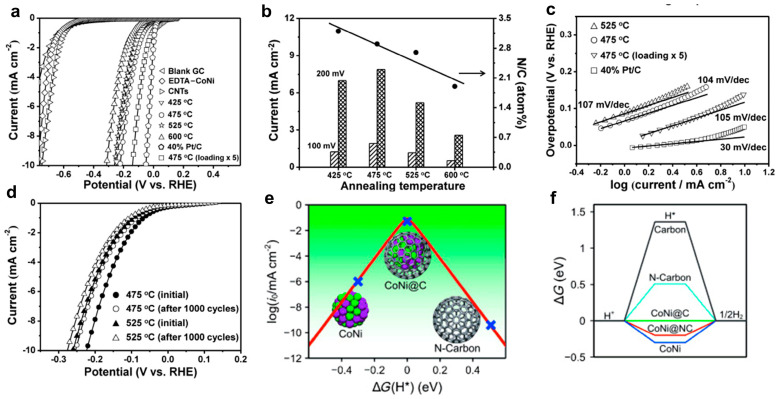
(**a**) LSV curves, (**b**) current density at overpotentials of 100 mV and 200mV, (**c**) Tafel plots, (**d**) durability of CoNi@NC catalysts with different thermal processing temperatures, (**e**,**f**) DFT analysis results [8]. Reproduced with permission from ref. [8]. Copyright 2015, Wiley.

Based on these experimental phenomena, Deng et al. [8,58] prepared ultra-thin graphene-coated cobalt-nickel alloys using EDTA complexation. The resulting graphene shells were observed to be only 1 to 3 layers, with over 90% of the graphene consisting of 1 to 2 layers. The optimal CoNi@C chainmail catalysts exhibited an η_10_ of 142 mV, which is comparable to that of commercial Pt/C (Figure 6). Asefa et al. [59] also reached the conclusion that the encapsulated metal nanoparticles are capable of reducing the localized work function on the surface of the carbon layer. This is achieved through the transfer of electrons from the metal particles to the graphene. Furthermore, the carbon layer can be coupled with N-dopants, which facilitate hydrogen adsorption and thus enhance HER performance. It has been demonstrated that the HER activity of transition metals with different coatings varies, with Fe exhibiting the lowest activity, followed by Ni and Co. In a recent development, the research group led by Wei Gao proposed a straightforward and expeditious method to synthesize nitrogen-doped carbon-coated high-entropy alloy nanoparticles with a tunable thickness of the carbon shell layer. It was observed that the HER of the alloys exhibited a gradual increase with the addition of alloying elements. The six-element high-entropy alloy CuNiFeCoCrTi@NC NPs demonstrated the most favorable HER performance and remarkable long-cycle stability. Meanwhile, the impact of a nitrogen-doped graphene shell layer on the HER performance of the alloy was investigated, demonstrating that the chainmail structure and an alloy nucleus effectively regulated ΔG_H_* [60]. Zhou et al. [61] designed N-doped Co@C chainmail catalysts, and found that the transfer of approximately 0.7 electrons from Co to neighboring carbon atoms resulted in an elevation of the Fermi energy level and the introduction of energy levels within the HOMO-lowest LUMO gap of the carbon layer. The obtained Co@NC/NG catalyst had an HER of η_10_ of only 49 mV (vs RHE) in 1 M KOH. 

### 3.2. Transition Metal Carbides Chainmail Catalysts 

TM carbides, especially molybdenum carbide, have long been expected, due to having d-electronic density of states (DOS) and high electrical conductivity similar to Pt, to be non-Pt HER electrocatalysts [62,63,64]. However, the HER performance of Mo_2_C is typically constrained by the presence of strong hydrogen binding energy, which in turn impedes the formation of water cleavage sites within the catalyst apparatus. To solve this, Asefa et al. [49] encapsulated molybdenum carbide nanoparticles in graphene shells (Mo_2_C@NC). The Mo_2_C@NC heterogeneous nano-catalysts exhibited superior HER catalytic activity and stability over pH of 0–14, and a Faraday efficiency of about 100%. DFT calculations demonstrated a synergistic interaction between the Mo_2_C layer and the chainmail through an electron transformation that modulated the adsorption energy of *H and *OH to a moderate level, thereby increasing the HER performance. Ma et al. [65] employed a metal–organic complex-assisted pyrolytic carbonization method to prepare an ultrafine Mo_2_C chainmail catalyst with outstanding HER performance. It was determined that the ultrathin graphene shell facilitated the electron transformation between the Mo_2_C and the chainmail surface, thereby enhancing the reactivity towards the HER.

**Figure 7:**
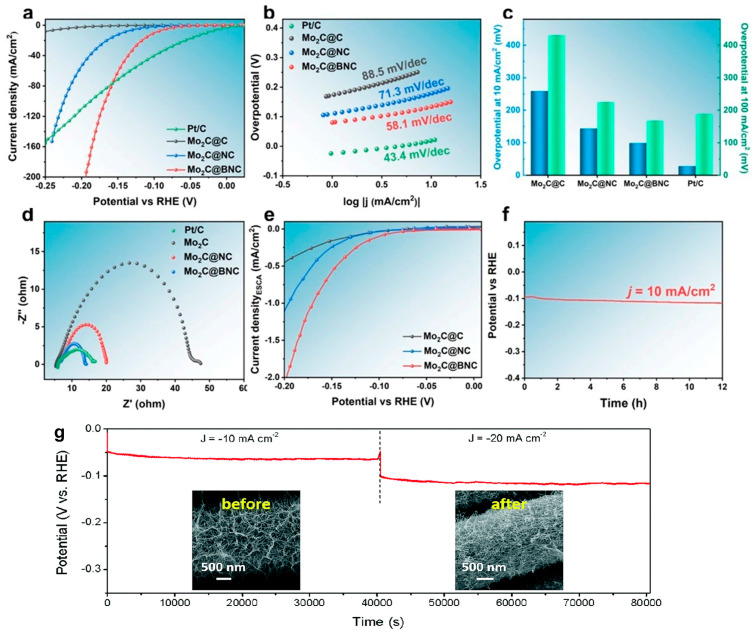
(**a**–**f**) (**a**) LSV curves; (**b**) Tafel slopes; (**c**) η_10_ and η_100_; (**d**) EIS curves; (**e**) polarization curves normalized by ECSA of Mo_2_C@C, Mo_2_C@NC, and Mo_2_C@BNC; (**f**) V-t curves of Mo_2_C@BNC [66], reproduced with permission from ref. [66], copyright 2023, Springer; (**g**) the stability test of MoO_2_-Ni@NC in 0.5 M H_2_SO_4_ [67], reproduced with permission from ref. [67], copyright 2018, Royal Society of Chemistry.

In order to further optimize HER activity, the research group led by Zhang et al. [66] prepared a B, N double-doped Mo_2_C@C chainmail catalyst for an alkaline HER. The double-doping of N and B on the surface of the chainmail layer resulted in the formation of multiple active centers, which led to a notable enhancement in HER activity. The catalyst exhibited excellent HER activity with only η_10_ of 99 mV (Figure 7a–f). Wu et al. [67] rationally chose polyoxomolybdate containing both Mo and Ni as the precursor for the preparation of carbon-coated multicomponent catalysts, prepared nitrogen-doped carbon-coated MoO_2_ and Ni ultrathin nanowire arrays with a diameter of ~15 nm by gradient pyrolysis, and found that the order of the HER activity was MoO_2_-Ni@NC > MoO_2_@NC > NC. They proposed that the carbon-coated multicomponent catalyst had superior HER catalytic activity compared with the carbon-coated single-component catalysts and attributed the excellent HER performance of MoO_2_-Ni@NC to the following aspects: (1) The internal MoO_2_ and Ni had a synergistic modulation effect on the N-doped chainmail surface. On the one hand, when MoO_2_ was introduced into the N-doped chainmail layer, it weakened H–N antibonding and enhanced surface *H adsorption; on the other hand, when the introduction of the Ni species was continued, the additional formation of H–C_2_ bonding further optimized the *H strength, thus enhancing the HER kinetics. (2) The multilayered carbon with a higher degree of graphitization slowed down the solubilization of metallic Ni (Figure 7g). And, theoretical analysis reveals that the active sites of N-doped carbon-coated catalysts are located on the C atoms next to N-doped atoms [68,69]. 

### 3.3. Noble Metal Doping: Transition Metal Chainmail Catalysts

In addition to non-precious metal TMs (Fe, Co, Ni, etc.), other noble metals (e.g., Pt, Ru) can also be used as additives to alloy with non-precious metals to be encapsulated within the carbon layer to enhance the HER activity. Xu et al. [13] prepared nitrogen-rich carbon-covered nickel-ruthenium alloys (NiRu@N-C) by loading a small amount of Ru through a nickel-based MOF material, followed by high-temperature calcination in nitrogen gas. The catalyst demonstrated promising HER activity and stability across a broad pH range. In acidic conditions, η_10_ was observed to be 50 mV with a Tafel slope of 36 mV/dec; under alkaline conditions, η_10_ was found to be 32 mV. It was shown that such high activity was due to the introduction of a certain amount of Ru, and the internal formation of NiRu alloy promoted electron transfer from the inner core to the graphene layer, which in turn improved its electrocatalytic activity. Su et al. [70] reported a RuCo@C chainmail catalyst. The overpotentials of this catalyst at 10 and 100 mA/cm² under alkaline conditions were 28 and 218 mV, respectively. Theoretical calculations indicated that doping Ru in the Co core improved the ability and efficiency of electron transfer from the inner metal core to the chainmail surface. This facilitated the strengthening of carbon-hydrogen bonding, thereby reducing the ΔG_H_* of the HER. Si et al. [71] developed a bifunctional catalyst by employing a urea-assisted pyrolysis process to create an N-doped IrCo@C chainmail catalyst. The strong interactions between Ir-, Co-, and N-doped chainmail resulted in significantly enhanced catalytic activities for both HERs and OERs. 

### 3.4. Transition Metal Phosphide-Based Chainmail Catalysts

Transition metal phosphide-based catalysts can also be employed as alternatives to Pt-based catalysts, exhibiting considerable potential for HERs. Zang et al. selected a nitrogen-phosphorus two-heteroatom hybrid copper-based metal-organic framework material (Cu-NPMOF) as a single-source precursor to prepare a novel Cu_3_P@NPPC catalyst. The multilevel pore structure of the nitrogen- and phosphorus-doped carbon matrix, in conjunction with the synergistic catalytic effect with cuprous phosphide nanoparticles, resulted in an outstanding HER electrocatalytic performance, characterized by an overpotential of 89 mV and a Tafel slope of 76 m [72]. A self-supported bifunctional catalyst (C@CoP-FeP/FF) with a carbon-coated bimetallic phosphide structure was prepared using a simple two-step method by Wang et al., and electrochemical tests showed that the overpotentials of the OER and HER of C@CoP-FeP/FF were 297 mV and 154 mV, respectively, at 100 mA cm^−2^. In addition, the catalyst also exhibited excellent catalytic activity and stability in a simulated seawater electrolyte [73]. The research group led by Cao et al. developed a method for producing HER electrocatalysts with vertically aligned core-shell structures (CoP/NPC/TF) on Ti sheets. This involved the use of CoP nano-arrays as cores and N- and P-doped carbon (NPC) as shells. The findings demonstrated that CoP/NPC/TF necessitated a mere 91 and 80 mV overpotential to attain a current density of 10 mA/cm² in acidic and alkaline solutions, respectively. Electrochemical tests and theoretical calculations demonstrated that the incorporation of porous NPC shells enhanced the availability of active sites and improved the conductivity and stability of the material in acidic and alkaline conditions. Furthermore, DFT calculations indicated that all the C atoms situated between the N and P atoms in the CoP/NPC were the most efficient active sites, which significantly optimized HER performance [74].

### 3.5. Carbon-Based Single Atom Catalysts

Carbon-based single-atom catalysts have been considered a special kind of carbon-coated chainmail catalyst due to their unique structural properties, which can achieve effective reactions, minimize catalyst costs, and promote the wider adoption of hydrogen evolution reactions (HERs) in industry [75]. Yin et al. employed a four-coordination C_2_-Pt-Cl_2_ species to construct and synthesize a carbon-based Pt single catalyst. The resulting Pt-single catalyst exhibited markedly elevated catalytic performance for the HER, with a superior mass activity of 26.9-fold over that of commercial Pt/C catalysts [76]. Wang et al. synthesized Ru atoms supported upon a nitrogen, sulfur-mixed graphene oxide (GO) catalyst using a hydrothermal approach, which demonstrated a stable HER performance. Ru single atoms establish strong interactions with N and S atoms, resulting in a strong contact between the Ru single atoms and the carrier. This robust coordination architecture efficiently enhanced the active center’s capacity to produce hydrogen and adsorb hydrogen ions, leading to superior HER activity for the carbon-supported Ru single-atom catalyst [77]. Furthermore, in order to improve HER performance, scientists have examined changing the active site architecture in carbon-based single-atom catalysts. Ashwani established a straightforward method to manufacture a bimetallic element, single-atom heterodimer of nickel and cobalt on N-doped carbon, which demonstrated remarkable HER performance with low overpotential. DFT analyses showed that the d-band centers were dramatically upshifted nearer to the Fermi level due to the beneficial relationship at the Ni-Co atomic interface [78].

## 4. Summary and Outlook

**Figure 8:**
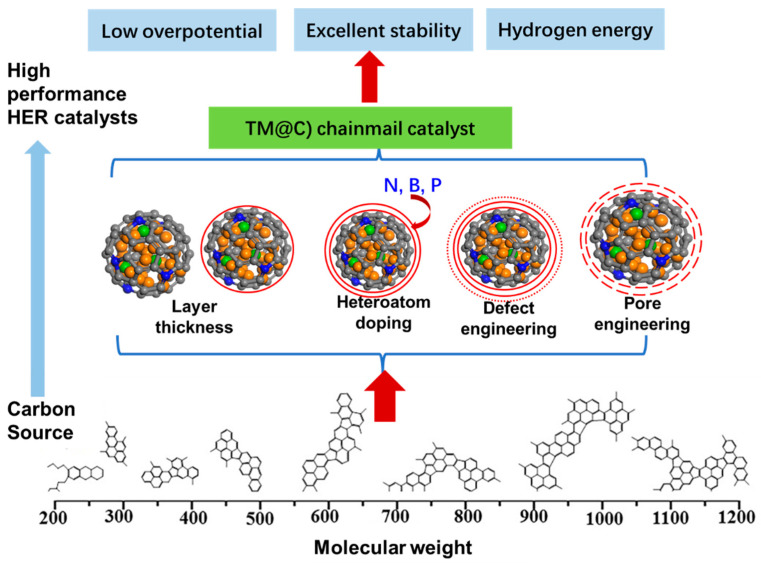
The main strategies for regulating the structure and electrons to optimize HER performance with a TM@C chainmail catalyst.

TM@C chainmail catalysts have allowed significant advancements in enhancing catalytic performance; however, there are still challenges to be addressed in practical applications. These include the following: (1) the pyrolysis process of the metal precursor is affected by various factors, which presents difficulties in the controlled synthesis of the “carbon chainmail”; (2) ensuring the electronic state coupling between the “chainmail layer” and the metal core is challenging, and the confusion of heteroatom doping and metal doping makes it difficult to fine-tune the catalyst. This review proposes the main strategies for regulating the structure and electrons to optimize HER performance, as illustrated in Figure 8. 

### 4.1. The Thickness of the Chainmail Layer

**Figure 9:**
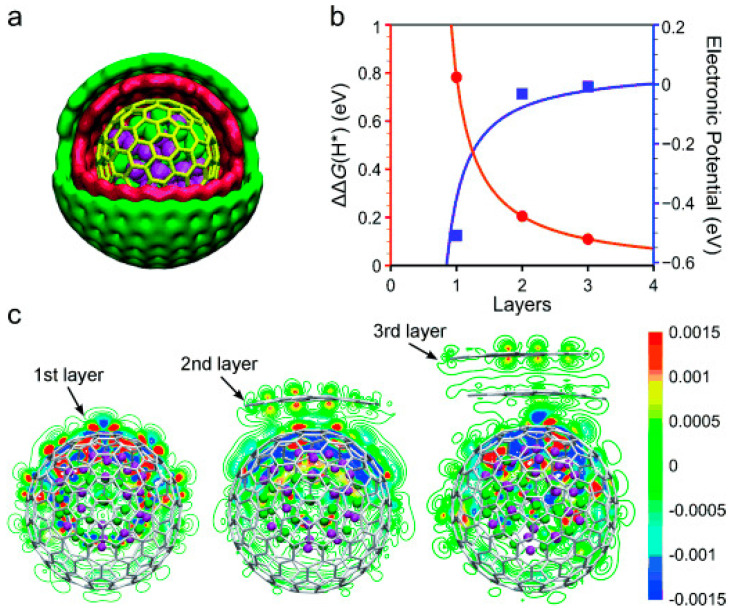
(**a**) The schema of CoNi@graphene, (**b**) variation of hydrogen adsorption energy with the number of layers, (**c**) the electron densities of CoNi@graphene with different layers [8]. Reproduced with permission from ref. [8]. Copyright 2015, Wiley.

The HER reactivity of chainmail catalysts originates from electron penetration into the chainmail layer through encapsulated metal particles, and the key to achieving this process lies in the modulation of the chainmail layer thickness [79,80]. Therefore, in the design of chainmail catalysts, the thickness of the carbon layer is crucial to optimize HER performance. The impact of a graphene-like carbon layer’s thickness on HER has been examined through a computational modeling approach (Figure 9). The results demonstrate that due to the increased electron density on the chainmail near the CoNi clusters, the stable adsorption of H* species is increased, which stimulates the HER activity of the chainmail layers. In addition, the electron penetration effect of the inner metal layer decreases with the number of armor layers for up to three carbon layers (Figure 2) [8,58]. Therefore, it can be concluded that the chainmail surface is difficult to activate with inner metals if the chainmail layer is >3. Consequently, a more optimal choice is to reduce the thickness of the chainmail layer to only one layer to enhance its transparency to the inner metals.

### 4.2. Heteroatom Doping in the Carbon Layer

The introduction of heteroatoms (such as N, B, and P) has been demonstrated to be an effective method for regulating the electronic and catalytic properties of graphene through the mediation of electronic interactions [81]. For instance, it has been suggested that N-doping facilitates electron transport from the inner Fe nanoparticles to the chainmail layers, thereby enhancing catalytic activity [49]. The co-doping of B and N can result in a synergistic effect concerning the regulation of the electronic properties of chainmail carbon atoms [82]. Furthermore, this approach can be combined with the encapsulation of metal nanoparticles, thereby promoting electrocatalytic hydrogen evolution activity. P-doping has the effect of increasing the electronegativity of graphite carbon, which in turn enhances the adsorption of H* [83]. In conclusion, heteroatom doping can be used in conjunction with transition metal nanoparticles to regulate the electronic properties of surface carbon atoms, optimize hydrogen adsorption energy, and improve hydrogen evolution performance. It can therefore be concluded that a rational choice of heteroatom doping, based on the electronic structure characteristics of the carbon layer and the core metal, represents a viable path to improving HER activity.

### 4.3. Defects Engineering

Defects engineering is an effective means to regulate the catalyst charge to enhance catalytic performance [84,85]. Firstly, defects directly serve as reaction active sites. When defects exist on the surface of the catalyst, the active atoms at the defect sites have an unsaturated coordination environment, which is more favorable for the adsorption of reactive molecules [66]. Secondly, defects can alter the local electronic structure or inject charges into sp2-hybridized carbon materials. Conversely, changes in electronic structure can directly influence the adsorption energies of reacting species with active sites, thereby affecting the kinetics and selectivity of catalytic reactions [86]. It has been noted that the volcano-type curves between the electrocatalytic HER performance and surface charge reveal the dependence of the catalytic reaction activities of HER of the carbon-based catalysts on the defects, and the catalytic activity is gradually enhanced with the increase in defects raising the enrichment of the surface charge, but when there are too many defects leading to high surface charge, the catalytic activity decreases along with it [87]. Therefore, defects change the electronic structure and play a decisive role in the change in catalytic activity, and such a dependence has been found in many studies of defective catalysts [88]. 

### 4.4. Pore Engineering: Multiple Active Site Designs

Porous nanostructures have a wide range of potential applications in the HER field due to their ability to enhance substance transport efficiency and increase the utilization of active sites during electrocatalytic reactions [89,90]. The construction of suitable porous nanostructures and the understanding of their constitutive relationships in energy electrocatalysis are important for the development of highly efficient electrocatalysts [91]. A straightforward account of the matter indicates that the active sites are located on the metal surface, while the chainmail layer serves to safeguard the nanoparticles [92]. Furthermore, it can provide an unconventional confined environment in which only specific chemicals can enter. Additionally, further consideration must be given to the sieving effect of the porosity of the chainmail layer on electrolytes and reaction products to optimize the reaction activity [93].

## Data Availability

The dataset is available upon request from the authors.

## Supplementary Materials

The following supporting information can be downloaded at: https://www.mdpi.com/article/10.3390/molecules29194677/s1, Table S1: Summary of TM@graphene catalysts for HER [94,95,96].
