# Supplementary material for: Encapsulating Transition Metal Nanoparticles inside Carbon (TM@C) Chainmail Catalysts for Hydrogen Evolution Reactions: A Review
Source: Molecules. 2024 Oct 2;29(19):4677. doi: 10.3390/molecules29194677 (PMC11477923; doi:10.3390/molecules29194677)
Supplement: Supplementary file 1 [file molecules-29-04677-s001.zip › molecules-3217176-supplementary.docx]

Supporting Information for

Encapsulating transition metal nanoparticles inside carbon (TM@C) chainmail catalyst during HER: A review

Jiamin Zhao^1^, Meimei Kou^1^, Qing Yuan^1^, Ying Yuan^1,*^, Jinsheng Zhao^1,*^

Table 1 Summary of TM@graphene catalysts for HER

| Catalysts | structure | 电解质 | 过电位  （10mA cm^-2^) | Tafal  （mV/decade） | ref |
| --- | --- | --- | --- | --- | --- |
| [CoNi@graphene](mailto:CoNi@graphene) | CoNi alloy encapsulated in ultrathin graphene shell (1–3 layers) | 0.1M H_2_SO4 | 142 | 104 | [1] |
| Mo_2_C@graphene | Ultrasmall Mo_2_C nanoparticle embedded within nitrogen-rich carbon nanolayers | pH=0 | 124 | 60 | [2] |
|  |  | pH=7 | 156 | / |  |
|  |  | pH=14 | 60 | / |  |
| CoMoO@graphene | Uniform CoMnO nanoparticles coated with a thin,  continuous nitrogen-doped carbon (CN) framework | 1 M KOH | 71 (20mA cm-2) | 152 | [3] |
| Co_2_P@graphene | Co_2_P encapsulated in N,P-doped graphene | 0.5 H2SO4 | 103 | 58 | [4] |
| Co@graphene | Metallic Co encapsulated in nitrogen-rich  carbon nitride | 0.5 H2SO4 | 200 | 82 | [5] |
| B,N-doped Mo2C@graphene | B, N double-doped ultrathin carbon layer capped Mo₂C nanocrystals | 1M KOH | 99 | 55.1 | [6] |
| MoO_2_-Ni@graphene | Nitrogen-doped carbon coated MoO2-Ni nanowire arrays | 0.5M H2SO4 | 58 | 35.1 | [7] |
| Ni/MoO_2_@C | Three-Phase Heterojunction NiMo-Based Nano-Needle | 1M KOH | 33 | 45 | [8] |
| CoP-FeP@ graphene | Bimetallic Phosphide Heterostructure Coupled with Ultrathin Carbon Layer | 1M KOH | 154(100mAcm-2) | 67.2 | [9] |
| NiFeCo@ graphene | NiFeCo-based High Entropy Alloys Nanoparticles Coated with N-doped Graphene Layers | 1.0 M KOH | 106 | 113 | [10] |

1. Deng, J.; Ren, P.; Deng, D.; Bao, X., Enhanced Electron Penetration through an Ultrathin Graphene Layer for Highly Efficient Catalysis of the Hydrogen Evolution Reaction. *Angew. Chem. Int. Ed.* **2015,** 54, (7), 2100-2104.

2. Liu, Y.; Yu, G.; Li, G.-D.; Sun, Y.; Asefa, T.; Chen, W.; Zou, X., Coupling Mo2C with Nitrogen-Rich Nanocarbon Leads to Efficient Hydrogen-Evolution Electrocatalytic Sites. *Angew. Chem. Int. Ed.* **2015,** 54, (37), 10752-10757.

3. Li, J.; Wang, Y.; Zhou, T.; Zhang, H.; Sun, X.; Tang, J.; Zhang, L.; Al-Enizi, A. M.; Yang, Z.; Zheng, G., Nanoparticle Superlattices as Efficient Bifunctional Electrocatalysts for Water Splitting. *J. Am. Chem. Soc.* **2015,** 137, (45), 14305-14312.

4. Zhuang, M.; Ou, X.; Dou, Y.; Zhang, L.; Zhang, Q.; Wu, R.; Ding, Y.; Shao, M.; Luo, Z., Polymer-Embedded Fabrication of Co_2_P Nanoparticles Encapsulated in N,P-Doped Graphene for Hydrogen Generation. *Nano Lett.* **2016,** 16, (7), 4691-4698.

5. Dai, X.; Li, Z.; Ma, Y.; Liu, M.; Du, K.; Su, H.; Zhuo, H.; Yu, L.; Sun, H.; Zhang, X., Metallic Cobalt Encapsulated in Bamboo-Like and Nitrogen-Rich Carbonitride Nanotubes for Hydrogen Evolution Reaction. *ACS Appl. Mater. Interfaces* **2016,** 8, (10), 6439-6448.

6. Zhou, M.; Jiang, X.; Kong, W.; Li, H.; Lu, F.; Zhou, X.; Zhang, Y., Synergistic Effect of Dual-Doped Carbon on Mo_2_C Nanocrystals Facilitates Alkaline Hydrogen Evolution. *Nano-Micro Lett.* **2023,** 15, (1), 166.

7. Liu, X.; Ni, K.; Wen, B.; Niu, C.; Meng, J.; Guo, R.; Li, Q.; Li, J.; Zhu, Y.; Wu, X.; Zhao, D.; Mai, L., Polyoxomolybdate-derived carbon-encapsulated multicomponent electrocatalysts for synergistically boosting hydrogen evolution. *J. Mater. Chem. A* **2018,** 6, (37), 17874-17881.

8. Qian, G.; Chen, J.; Yu, T.; Liu, J.; Luo, L.; Yin, S., Three-Phase Heterojunction NiMo-Based Nano-Needle for Water Splitting at Industrial Alkaline Condition. *Nano-Micro Lett.* **2021,** 14, (1), 20.

9. Li, J.; Hu, Y.; Huang, X.; Zhu, Y.; Wang, D., Bimetallic Phosphide Heterostructure Coupled with Ultrathin Carbon Layer Boosting Overall Alkaline Water and Seawater Splitting. *Small* **2023,** 19, (20), 2206533.

10. Gao, Q.; Wang, Z.; Gao, W.; Yin, H., NiFeCo-based high entropy alloys nanoparticles coated with N-doped graphene layers as hydrogen evolution catalyst in alkaline solution. *Chem. Eng. J.* **2024,** 489, 151370.
